# Precision neurodiversity: personalized brain network architecture as a window into cognitive variability

**DOI:** 10.3389/fnhum.2025.1669431

**Published:** 2025-11-12

**Authors:** Suleiman Ibrahim Mohammad, Eman Raeed Azzam, Asokan Vasudevan, Sayed M. Ismail, Hina Ayaz, K. D. V. Prasad

**Affiliations:** 1 Electronic Marketing and Social Media, Economic and Administrative Sciences Zarqa University, Zarqa, Jordan; 2 INTI International University, Nilai, Negeri Sembilan, Malaysia; 3 Medical Laboratory Techniques Department, College of Health and Medical Technology, University of Al-maarif, Anbar, Iraq; 4 Faculty of Business and Communications, INTI International University, Nilai, Negeri Sembilan, Malaysia; 5 Shinawatra University, Pathum Thani, Thailand; 6 Department of English Language and Literature, College of Science and Humanities, Prince Sattam Bin Abdulaziz University, Al-kharj, Saudi Arabia; 7 Department of Psychology, Hamdard University, Karachi, Pakistan; 8 Symbiosis Institute of Business Management, Hyderabad, India; 9 Symbiosis International (Deemed University), Pune, India

**Keywords:** brain connectome, neurodiversity, precision medicine, magnetic resonance imaging, machine learning, neurodevelopmental disorders

## Abstract

Precision neurodiversity marks a shift in neuroscience from pathological models to personalized frameworks that view neurological differences as adaptive variations. This review synthesizes current knowledge on the Personalized Brain Network architecture and its relationship with cognitive variability in both typical and neurodiverse populations. The study examines advancements in connectome-based prediction modeling, normative modeling, dynamic fingerprinting, and machine learning methods that characterize individual-specific neural networks. Recent findings indicate that the Personalized Brain Network profile reliably predicts cognitive, behavioral, and sensory phenomena. Additionally, deep generative models demonstrate high fidelity in synthesizing connective cells. Recent studies have identified distinct neurobiological subgroups in conditions such as attention-deficit hyperactivity disorder (ADHD) and autism spectrum disorder that were previously undetectable by conventional diagnostic criteria. However, research has revealed significant network-level differences among these subgroups. Researchers have identified age-resistant biomarkers in specific brain regions, and genetic mutations significantly influence the connectivity patterns of individuals. Clinical applications span a range of neurodevelopmental conditions, including autism, ADHD, dyslexia, and talent. Network variability predicts executive functioning, social perception, and sensory processing abilities. However, successful translation requires overcoming challenges related to statistical power, reproducibility, ethical implementation and community participation. The convergence of advanced neuroimaging, artificial intelligence, and personalized medicine offers unprecedented opportunities for tailored interventions, while celebrating neurological diversity as a source of human strength.

## 1 Introduction

The current landscape of neuroscience has shifted from pathological deficit models to variation-based frameworks. This new approach views neurological differences as a natural manifestation of human brain diversity (Miranda-Ojeda et al., 2025; Ramanan et al., 2025). This transformation, termed precision neurodiversity, represents the convergence of two key concepts: the neurodiversity movement’s redefinition of neuro-conditions as adaptive variations and precision medicine’s commitment to an individual- and data-driven understanding of human biology (McGorry et al., 2025). Dimensional ones are increasingly replacing conventional categorical diagnostic frameworks. This shift reflects the growing evidence that traditional neurological boundaries fail to capture the complex and continuous nature of cognitive and neural variations (Leadbitter et al., 2021; Dwyer, 2022; Mardinoglu et al., 2025). Recent transdiagnostic studies have shown that dimensional models are more effective in identifying meaningful therapeutic targets across neurodevelopmental conditions (Madericova and Talcott, 2025; Alehagen et al., 2025).

The advent of population-scale neuroimaging studies has precipitated a paradigm shift in our understanding of neurodevelopmental disorders. Using standardized brain charts derived from over 123,000 structural MRI scans, researchers have identified distinct neurobiological subtypes within populations with ADHD. Two conditions have been identified: delayed brain growth (DBG-ADHD) and prenatal brain growth (PBG-ADHD). These conditions are not identifiable using conventional diagnostic criteria; however, they exhibit significant disparities in functional organization at the network level (Bu et al., 2024; Pecci-Terroba et al., 2025). These subtypes are indicative of distinct neurodevelopmental trajectories, with PBG-ADHD characterized by accelerated cortical growth patterns that can be detected using normative brain charts derived from large-scale structural MRI data.

The extent and conceptualization of neurodiversity within the precision medicine framework require careful theoretical clarification, especially given the ongoing scholarly debates regarding its boundaries (May, 2025; Van Daalen et al., 2025). We adopted a dimensional neurodiversity framework that distinguishes between core neurodevelopmental conditions—marked by early onset, pervasive patterns of atypical brain organization from childhood—and acquired or episodic psychiatric conditions. This framework is designed to encompass a range of neurodevelopmental and learning-related conditions, including Autism Spectrum Disorder (ASD), Attention-Deficit/Hyperactivity Disorder (ADHD), specific learning differences (e.g., dyslexia and dyscalculia), and intellectual disabilities. Including these conditions aims to provide a comprehensive overview of the spectrum of neurodevelopmental and learning-related challenges (Hunt and Procyshyn, 2024). While recognizing the valuable perspectives of individuals with conditions such as schizophrenia and bipolar disorder, we maintain a theoretical distinction due to their fundamentally different trajectories. These conditions typically have clear onset periods, show fluctuating or progressive courses, and involve distinct pathophysiological mechanisms compared to core neurodevelopmental variations (Miranda-Ojeda et al., 2025). This distinction does not undermine the importance of personalized approaches for all neurological conditions; instead, it acknowledges that different frameworks may be more suitable for understanding developmental versus acquired brain differences within precision medicine paradigms.

The conceptual foundations of the Personalized Brain Network architecture range from early neural attempts to map individual cognitive functions to contemporary connectomics methods that characterize whole-brain connectivity patterns at the single-subject level (Agnati et al., 2023). High-resolution functional Magnetic Resonance Imaging and advanced computational methods have enabled the identification of a specific “neural fingerprint” in individuals—a unique pattern of brain connectivity that remains stable over time, across tasks, and during aging processes (Lee and Lee, 2024; Zhang et al., 2024). Recent advances in deep generative modeling have enabled the inference of personalized human brain connectivity patterns from individual characteristics alone. Liu Y. et al. (2025) demonstrated that conditional variational autoencoders can generate human connectomes with remarkable fidelity using the UK Biobank dataset (*N* = 8,086), revealing that age, sex, and body phenotypes contribute approximately four times more to connectivity architecture than cognitive or lifestyle factors (Udayakumar and Subhashini, 2025). This breakthrough represents a fundamental shift from traditional group-level analyses to truly personalized brain network characterization, enabling precision approaches to neurodiversity. Methodological advances in human brain generative modeling have witnessed remarkable progress in human-specific generative models. Recent work by Seiler and Ritter (2025) highlights how generative brain networks enable the creation of virtual brain twins, integrating structural connectivity information into probabilistic frameworks specifically designed for human neurological disease research. Furthermore, Wu et al. (2024) demonstrated that generative adversarial networks can refine human brain structural connectivity strength while maintaining individual differences.

These advances have direct clinical implications for precision neurodiversity approaches. The ability to generate personalized human connectomes enables data augmentation for machine learning models, anonymous data sharing, and prediction of individual therapeutic responses—all critical components for implementing precision medicine in neurodevelopmental conditions. The stability of individual-specific brain signatures across the adult lifespan (ages 18–87) provides compelling evidence for core neuroanatomical characteristics that persist despite normal aging processes. Leverage score-based feature selection methods have identified specific brain regions that constitute age-resilient biomarkers of intrinsic brain organization (Taimouri and Ravindra, 2025; Zamani et al., 2022; Taimouri and Ravindra, 2025).

This comprehensive review synthesizes the current knowledge on Personalized Brain Network architecture and its relationship with cognitive variability within the precision neurodiversity framework. Our analysis encompasses three primary objectives: first, to critically evaluate methodological approaches for characterizing individual-specific neural networks; second, to examine how Precision Brain Network (PBN) architectures relate to cognitive phenotypes across neurotypical and neurodivergent populations; and third, to identify promising research directions that leverage precision neuroscience approaches to advance individualized interventions and support systems.

**Figure 1:**
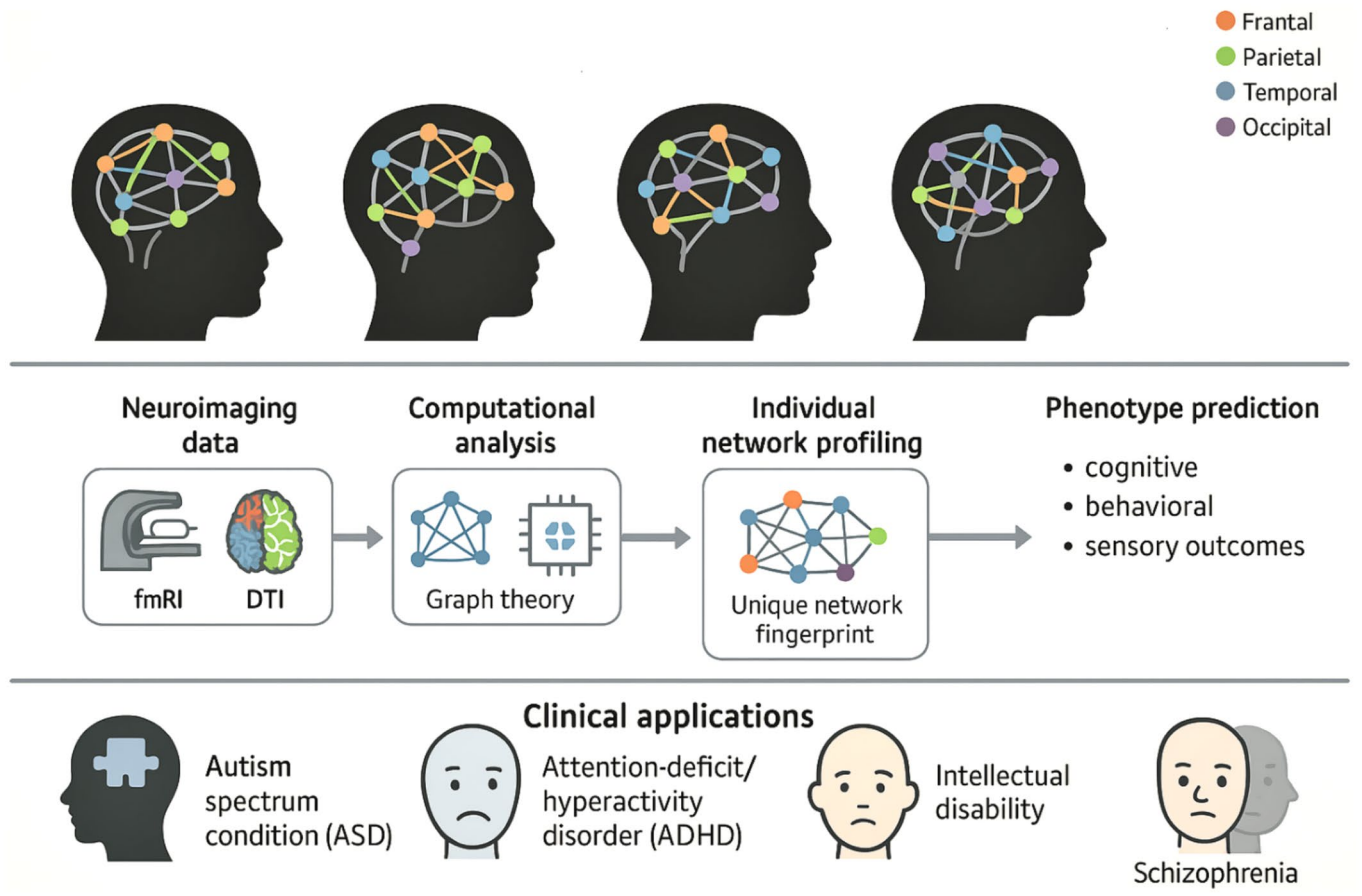
Conceptual framework of precision neurodiversity through personalized brain networks.

The comprehensive framework for implementing precision neurodiversity through Personalized Brain Network analysis is illustrated in Figure 1, which demonstrates the paradigmatic shift from categorical diagnostic approaches to individualized characterization of neurological diversity. This conceptual framework encompasses the complete workflow from neuroimaging data acquisition through computational analysis to clinical translation, emphasizing how each individual’s unique “neural fingerprint” can inform personalized interventions for major neurodevelopmental conditions.

This figure illustrates the comprehensive workflow for applying precision neurodiversity through Personalized Brain Network analysis, highlighting how individual neurological differences can be characterized as adaptive variations rather than pathological ones. The top panel displays four brain symbols representing different individual brain network architectures, each with unique connections between color-coded brain regions (frontal: orange, parietal: green, temporal: blue, occipital: purple), illustrating the concept of neurological diversity, where each person has a unique “neural fingerprint.” The middle panel illustrates the methodological workflow that progresses through the acquisition of neuroimaging data (fMRI and DTI), computational analysis using graph theory, extraction of individual network profiles to identify unique network characteristics, and phenotype prediction, which enables accurate predictions of cognitive, behavioral, and sensory outcomes. The lower panel shows clinical applications for major neurodevelopmental diseases, such as autism spectrum disorder (ASD), ADHD, intellectual impairment, and schizophrenia, which represent a paradigm shift toward individualized, data-based approaches that celebrate neurodiversity while enabling accurate, personalized interventions based on the unique structure of each individual’s brain network.

## 2 Theoretical foundations

### 2.1 Network neuroscience foundations

The mathematical framework of graph theory has become essential for describing the organization of brain networks, providing quantitative tools to describe complex patterns of neural connectivity and their relationship with cognitive function (Ni et al., 2025; Tuan et al., 2025). The brain network can be characterized as a node (brain region) and edge (regional connection). It can be systematically analyzed using metrics such as the cluster coefficient, path length, and centrality. Contemporary brain network analysis methods increasingly recognize that traditional group-level analysis can obscure critical individual differences in network organization (Amato et al., 2025; Fekonja et al., 2025). PBN neural mass models, with their potential to reveal specific patterns of network dysfunction associated with cognitive results, offer promising prospects for brain network analysis (Ye et al., 2024).

The brain network has topological characteristics that reflect the evolutionary optimization of the information processing efficiency. The characteristics of a small-world network, characterized by high clustering and short path lengths, are consistent across all neuroimaging methods and support local and global integration (Yang et al., 2025b; Shaheen et al., 2023). These architectures allow efficient information transfer while maintaining robustness to local disturbances. This field has evolved beyond the static concepts of brain connectivity, developing dynamic network frameworks that capture the temporal variations of functional relationships (Wen et al., 2025; Luijendijk et al., 2022). Dynamic connectivity approaches have demonstrated that brain networks are constantly reorganized across multiple timescales, ranging from rapid changes associated with tasks to slower developmental and pathological processes (Xin et al., 2022).

### 2.2 Individual differences in brain network organization

Genetic factors contribute significantly to individual differences in the structure of brain networks, and recent large-scale genomic studies have revealed complex inheritance patterns in brain regions and their network properties (Carrion-Castillo and Boeckx, 2024). A comprehensive analysis of the cerebellar substructures revealed that the inheritance rates in different regions ranged from 0.08 to 0.35, with most exhibiting moderate inheritance. Genome structural equation modeling indicates that brain regions are grouped into genetically distinct factors (Shishikura et al., 2025; Tissink et al., 2022). Environmental factors interact with genetic predispositions to shape individual network pathways throughout development and life (Zhou Q. et al., 2025; Xin et al., 2025a). Reading-related cognitive and brain features exhibit moderate heritability but are significantly influenced by education and language experience (Procopio et al., 2025; Zhao et al., 2023). This interaction between genes and the environment suggests that precision approaches to neurodiversity must consider both genetic predispositions and environmental factors when determining the characteristics of individual brain networks (Bao et al., 2025).

The development of the brain network follows characteristic paths that differ significantly from person to person, with early sensory-motor differences being the foundational elements of later cognitive and behavioral patterns. Contemporary neuroscience research indicates that sensory and movement differences often emerge as the initial manifestation of neurodiversity, preceding and potentially contributing to later differences in social communication and cognitive function (Kapp, 2025).

### 2.3 Cognitive variability: from pathology to diversity

Conventional wisdom regarding cognitive differences is primarily shaped by deficit-based models that view variations as deviations from the established norms. However, contemporary neuroscience challenges these frameworks by presenting cognitive differences as valid forms of human variation rather than as inherent disorders (Wang F. et al., 2025; Liu W. et al., 2025). This paradigm shift acknowledges that behavioral patterns do not necessarily reflect individual intentions and may be misinterpreted when viewed solely through neurotypical frameworks (Strock et al., 2025). Increasingly, contemporary approaches adopt dimensional models that continuously characterize cognitive function instead of categorizing typical and atypical functioning (Perl et al., 2025). These models suggest that cognitive abilities exist on a spectrum, with individuals displaying unique strengths and challenges in different areas. Network-based dimensional models show that cognitive functions arise from dynamic interactions among distributed brain systems rather than localized processing modules (Perl et al., 2025).

### 2.4 Precision medicine integration

The integration of precision medicine and neuroscience research requires robust strategies for the identification and validation of biomarkers. These strategies must capture individual differences in brain network organization and their correlations with cognitive outcomes (Bajinka et al., 2025). Recent advancements in neuroimaging analysis have shown that topological deviation indices can quantify individual network reorganization patterns and predict cognitive performance with high sensitivity (Ranasinghe and Mapa, 2024; Zhou Z. et al., 2025). Precision neurodiversity approaches facilitate the development of interventions tailored to individual brain network organization and cognitive function (Lessi et al., 2026). PBN models can identify specific pathological and physiological mechanisms contributing to network malfunctions, enabling the development of targeted therapeutic approaches (Wan et al., 2025; Rahman et al., 2023). This paradigm shift represents a transition from universally applicable interventions to customized medical approaches that optimize outcomes by aligning with the unique neuronal characteristics of individuals. Implementation of precise medicine methodologies in the context of neurodiversity raises significant ethical concerns, including consent, privacy, stigma, and potential discrimination based on neurobiological profiles (Maspul and Ardhin, 2025). Participatory research methodologies, which involve neurodiverse individuals as collaborators in research conceptualization and analysis, are essential for ensuring ethical conduct and community relevance (Paul Okugo et al., 2025).

### 2.5 Theoretical framework for neurodiversity scope

Our precision neurodiversity framework operates on three core principles that address ongoing debates regarding neurodiversity boundaries (May, 2025). First, developmental continuity: the included conditions show observable precursors in early development, with brain network differences detectable through normative modeling by school age (Bethlehem et al., 2022). Second, the stability of neural signatures: core network architectures remain identifiable across developmental stages, even as compensatory mechanisms may mask behavioral presentations (Vedechkina et al., 2025). Third, dimensional representation: conditions exist on continuous spectra rather than discrete categories, supporting individualized network profiling approaches (Posani et al., 2025). This framework explicitly excludes conditions with episodic onset patterns (e.g., major depressive episodes and acute psychotic episodes) and progressive deterioration (e.g., dementia and neurodegenerative diseases) while asserting that all neurological conditions deserve respectful, evidence-based care. This distinction acknowledges that neurodevelopmental conditions represent adaptive variations in brain organization present from early life, whereas other psychiatric conditions may involve disruptions in previously typical brain function (Cainelli and Bisiacchi, 2022). This theoretical position aligns with contemporary neurodiversity scholarship, emphasizing the importance of analyzing developmental trajectories in precision medicine applications (Hunt and Procyshyn, 2024).

## 3 Methodological approaches

### 3.1 Neuroimaging techniques for network characterization

Functional magnetic resonance imaging (fMRI) underpins PBN analysis and has undergone significant methodological advancements in recent years (Yen et al., 2023). Advanced methods use non-negative matrix factorization to clarify individual differences in cortical organization across various spatial scales, from coarse resolution (two networks) to acceptable resolution (30 networks) (Han et al., 2023; Wen et al., 2023). This approach employs group consensus rules to maintain cross-individual correspondence and data area rules to ensure spatial coherence (Han et al., 2025). Recent developments have focused on surface processing pipelines that retain individual cortical geometries (Goncalves et al., 2025). Enhanced preprocessing protocols utilize a complex motion correction strategy, including a 36-parameter differential regression model and a temporal filter (0.01–0.08 Hz), to improve signal quality while preserving individual differences (Mullin et al., 2025). Deep neural network architectures specifically designed to identify individuals from resting-state functional connections have shown that PBN fingerprints can be extracted with remarkable precision (Lee and Lee, 2024). The landscape of personalized brain network analysis features a range of complementary approaches, each providing unique advantages in identifying individual differences in brain organization (Kong and Jin, 2025; Karimi et al., 2025). Table 1 presents a comprehensive overview of the current methods, including neuroimaging techniques, computational methods, analytical frameworks, and validation strategies. This section outlines the main features, limitations, and recent advancements in this field.

**Table 1:** Methodological approaches for personalized brain network analysis.

Category	Method/approach	Technical description	Key advantages	Primary limitations	Sample size recommendations	References
Neuroimaging Techniques	Functional MRI (fMRI)	Measures blood oxygen level-dependent (BOLD) signals to map functional connectivity patterns. Advanced preprocessing includes 36-parameter confound regression, temporal filtering (0.01–0.08 Hz), and multi-echo denoising for improved signal-to-noise ratio.	High spatial resolution Non-invasive whole-brain coverage Established protocols Cross-site compatibility	Low temporal resolution Motion artifacts Indirect neural measure Scanner variability	*n* ≥ 200 for individual differences *n* ≥ 500 for multivariate models	Mullin et al. (2025)
	Diffusion Tensor Imaging (DTI)	Measures white matter microstructure and structural connectivity using water diffusion patterns. Advanced models include NODDI and free-water elimination for improved tissue specificity.	Direct structural assessment Tract-specific analysis Developmental sensitivity Clinical relevance	Complex acquisition requirements Processing intensive Motion sensitive Limited crossing fiber resolution	*n* ≥ 150 for tract analysis *n* ≥ 300 for connectome studies	Parker et al. (2025) and Liou et al. (2025)
	Multi-echo fMRI	Acquires multiple echo times to separate BOLD signal from non-neural noise sources. Enables biophysically-informed denoising and improved signal detection in problematic brain regions.	Superior denoising Improved ventral brain coverage Better reliability Reduced dropout artifacts	Longer acquisition time Complex preprocessing Limited availability Higher computational demands	*n* ≥ 100 for individual differences *n* ≥ 250 for network analysis	Constable et al. (2025)
Computational Methods	Graph Theory Analysis	Quantifies network topology using mathematical graph properties including clustering coefficient, path length, modularity, and hub identification. Enables characterization of network efficiency and organization.	Quantitative network metrics Cross-species compatibility Clinical interpretability Multi-scale analysis	Threshold dependency Resolution limits Null model assumptions Multiple comparisons	*n* ≥ 100 for basic metrics *n* ≥ 200 for individual differences	Yang et al. (2025a) and Ogut (2025)
	Machine Learning Approaches	Applies supervised and unsupervised algorithms including support vector machines, random forests, and deep neural networks for pattern classification and prediction of cognitive phenotypes.	High prediction accuracy Feature selection capability Non-linear relationships Automated analysis	Black box interpretability Overfitting risk Large sample requirements Hyperparameter sensitivity	*n* ≥ 300 for classification *n* ≥ 500 for deep learning	Raza et al. (2025) and Sharma and Chariar (2024)
	Connectome-based Predictive Modeling	Uses whole-brain functional connectivity patterns to predict behavioral and cognitive outcomes through feature selection and cross-validated Machine Learningframeworks.	Whole-brain integration Cross-validated predictions Individual-level precision Clinical translation potential	High dimensionality Feature interpretation challenges Site effects Generalizability concerns	*n* ≥ 200 for basic models *n* ≥ 400 for robust predictions	Ben-Zion et al. (2025) and Friedrich et al. (2024)
	Deep Generative Models	Employs variational autoencoders and generative adversarial networks to synthesize individual-specific connectomes and identify latent network representations underlying cognitive diversity.	Latent representation learning Data augmentation capability Unsupervised discovery Synthetic data generation	Training instability Computational complexity Validation challenges Limited interpretability	*n* ≥ 500 for training *n* ≥ 1,000 for robust models	Orlichenko et al. (2025) and Zhao (2025)
Analysis Frameworks	Individualized Parcellation	Optimizes brain parcel boundaries for each participant using methods like Group Prior Individualized Parcellation (GPIP) and Multi-Session Hierarchical Bayesian Modeling (MS-HBM).	Person-specific boundaries Improved functional alignment Enhanced effect sizes Cross-session stability	Computational intensive Multiple session requirements Method comparison challenges Validation complexity	*n* ≥ 50 per individual *n* ≥ 100 for group studies	DeYoung et al. (2025)
	Hyperalignment	Identifies corresponding functional units across individuals by aligning response patterns rather than anatomical landmarks, enabling improved cross-subject comparisons.	Functional correspondence Large effect sizes Cross-individual alignment Task-specific optimization	Task dependency Computational demands Limited to functional data Validation requirements	*n* ≥ 20 for alignment *n* ≥ 100 for generalization	Zhang et al. (2025a) and Li C. et al. (2025)
	Multimodal Integration	Combines structural, functional, and diffusion MRI data using joint dimensionality reduction, canonical correlation analysis, and multiview learning approaches.	Comprehensive brain characterization Improved prediction accuracy Cross-modal validation Rich phenotyping	Data alignment challenges Increased complexity Missing data issues Interpretation difficulties	*n* ≥ 150 for each modality *n* ≥ 300 for integration	Baghdadi et al. (2025) and Zhou R. et al. (2023)
Validation Approaches	Cross-validation	Employs k-fold, leave-one-out, and nested cross-validation strategies to assess model generalizability and prevent overfitting in predictive analyses.	Overfitting prevention Generalizability assessment Model selection guidance Statistical robustness	Reduced effective sample size Computational overhead Strategy selection challenges Bias-variance tradeoffs	*n* ≥ 100 for k-fold *n* ≥ 200 for nested CV	Jafrasteh et al. (2025) and Wang B. et al. (2025)
	External Validation	Tests model performance in completely independent datasets to assess true generalizability across sites, populations, and acquisition parameters.	True generalizability test Site effect assessment Population validity Clinical translation readiness	Requires multiple datasets Coordinate challenges Population differences Technical harmonization needs	Training: *n* ≥ 200 Validation: *n* ≥ 100	Xin et al. (2025b) and Jahanshad et al. (2024)
	Longitudinal Validation	Assesses temporal stability of individual network features across development and aging, revealing both stable and dynamic components of brain organization.	Temporal stability assessment Developmental insights Intervention timing guidance Biomarker identification	Long-term data collection Participant retention challenges Developmental confounds Practice effects	*n* ≥ 100 baseline *n* ≥ 2 timepoints minimum	Vidal-Piñeiro et al. (2025) and Boudreau et al. (2025)

Sample size recommendations: Based on power analyses for 80% power to detect medium effect sizes (r ≥ 0.30) with α = 0.05. Larger samples recommended for smaller expected effects or more complex analyses. CPM: Connectome-based Predictive Modeling; DTI: Diffusion Tensor Imaging; EEG: Electroencephalography; FA: Fractional Anisotropy; fMRI: functional Magnetic Resonance Imaging; GCN: Graph Convolutional Network; iFOD2: second-order integration over fiber orientation distributions; MD: Mean Diffusivity; MEG: Magnetoencephalography; ML: Machine Learning; MRI: Magnetic Resonance Imaging; t-SNE: t-distributed Stochastic Neighbor Embedding; UMAP: Uniform Manifold Approximation and Projection.

This methodological diversity reflects the complex nature of individual brain network characterization, which requires the integration of multiple approaches to capture personalized neural signatures fully.

The methodology of structural connectivity mapping through diffusion MRI has evolved significantly, moving from traditional Diffusion Tensor Imaging (DTI) to more advanced multi-shell and multi-tissue modeling techniques. The use of advanced tractography algorithms, such as the iFOD2 (second-order integration over fiber orientation distributions) method, enables more accurate white matter tract reconstruction by accommodating complex fiber geometries and crossing configurations (Yeh et al., 2021).

The integration of multiple neuroimaging modalities represents a key advancement in PBN analysis. Recent progress has demonstrated the combined benefits of merging high spatial resolution fMRI with high temporal resolution EEG/MEG to capture both complex anatomical network organization and millisecond-scale temporal dynamics. Innovative approaches employ simultaneous electroencephalogram (EEG)-fMRI acquisition protocols to map individual differences in connectivity strength and temporal coupling patterns across brain networks (Mantini et al., 2010; Jacobs et al., 2014).

### 3.2 Computational methods for individual network profiling

Graph-theoretical approaches have significantly evolved to address the complexities of PBN analysis. Contemporary frameworks go beyond simple global metrics to characterize individual differences in hierarchical network organization, focusing on multi-scale modularity and core-periphery structures (Pines et al., 2022). Advanced centrality measures, such as eigenvector centrality, betweenness centrality, and participation coefficient analyses, provide detailed characterizations of individual node-level network roles (Wang Y. et al., 2025; Ni et al., 2025).

The application of ML techniques to PBN analysis has dramatically expanded, with novel architectures designed explicitly for neuroimaging data (Qin et al., 2025). Deep learning approaches, particularly GCNs and attention-based models, enable the extraction of complex nonlinear relationships within individual connectivity patterns that cannot be captured by traditional linear methods (Sounthararajah et al., 2025; Zhou Y. et al., 2023). Multi-scale functional connectivity approaches using hierarchical graph convolutional networks predict individual behavior by integrating connectivity information across multiple spatial scales (Liu et al., 2024).

Contemporary dimensionality reduction methods for PBNs have progressed beyond traditional principal component analysis to more sophisticated nonlinear techniques that can capture complex individual differences (You et al., 2025; Runfola et al., 2025). Advanced manifold learning techniques, including t-SNE, UMAP, and autoencoders, demonstrate that individual brain networks occupy distinct positions within low-dimensional representational spaces (Lang et al., 2024; Poologaindran et al., 2025). Nonlinear methods like t-SNE and UMAP specialize in extracting manifold structures from PBNs. However, since both techniques are distance-preserving embeddings, noise in sparse connectome data can be amplified, leading to artifactual clustering. This can occur if the clustering process takes place far from the actual representations of neurodiversity. The use of counterfactual techniques within predictive frameworks like CPM may further complicate matters by conflating descriptive geometry with causal inference. Simulations show that individual-level predictions may have a variance inflation of up to 30% compared to the actual data, attributed to the omission of temporal processes within the model (Drysdale et al., 2017). Using population centroids as a reference is crucial in normative modeling methods, as it effectively addresses the aforementioned issue. This approach enhances their ability to detect subtle variations in neurodivergent profiles, although it is less sensitive to rare variants compared to other methods.

### 3.3 Validation and generalizability assessment

**Figure 2:**
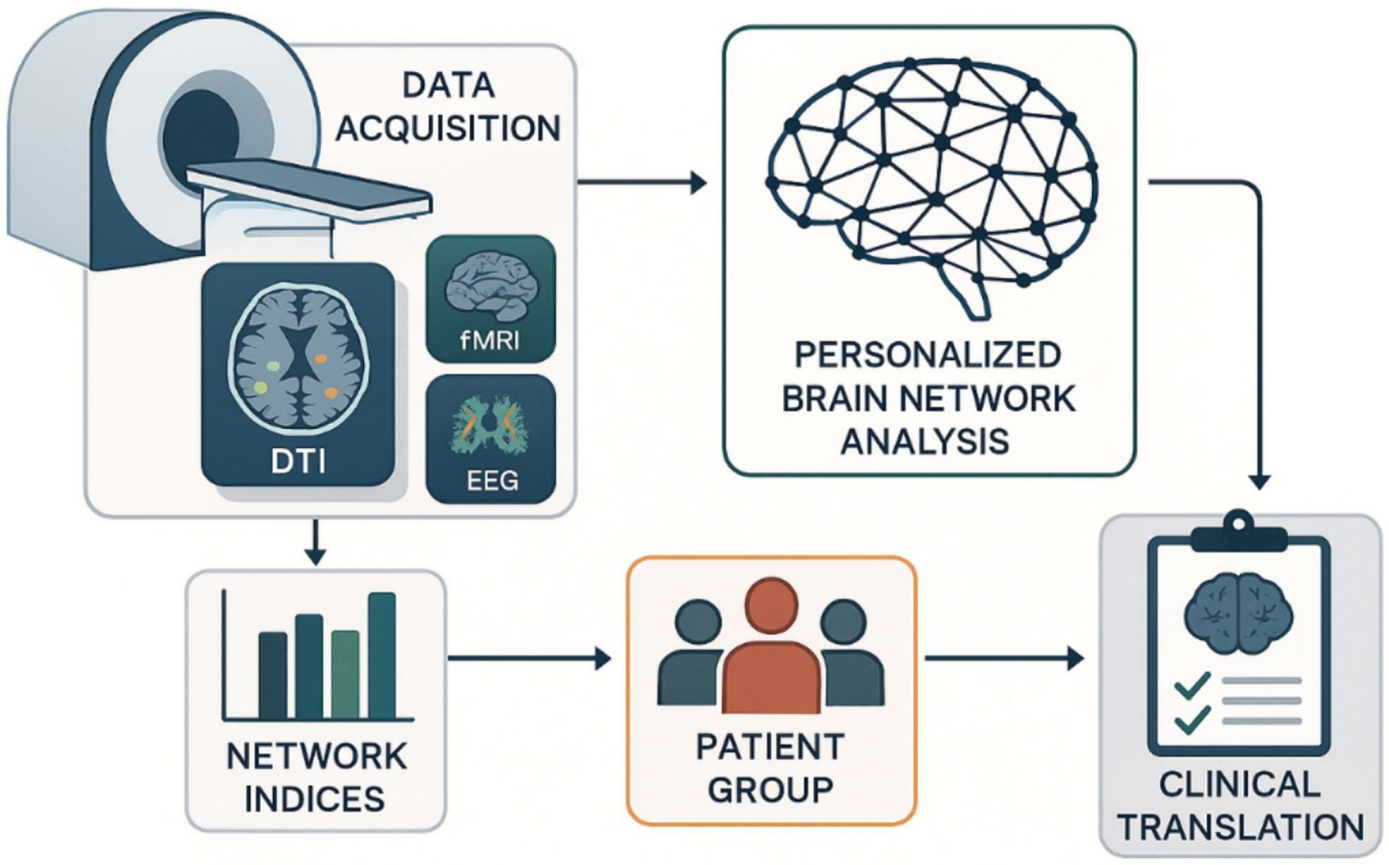
Methodological pipeline and clinical translation of personalized brain network analysis.

Given the complexity and potential overfitting of individual difference patterns, a robust validation framework is essential for PBN analysis. Contemporary approaches focus on embedded cross-validation strategies that optimize model hyperparameters separately and assess generalization performance on independent datasets (Parvandeh et al., 2020; Lewis et al., 2023). One-site testing strategies are increasingly recognized as vital for evaluating the generality of PBN models across different scanners and acquisition protocols (Friedman et al., 2008). The longitudinal validation method assesses the temporal stability of individual network functions, demonstrating that PBN architectures exhibit stable and dynamic components throughout development and aging (Wright et al., 2016). These findings have important implications for determining when targeted interventions are most effective. Translating individualized brain network analysis from research to clinical practice requires a systematic pipeline that integrates multimodal neuroimaging data with computational network analysis and clinical symptoms (Lewis et al., 2023). Figure 2 illustrates this comprehensive methodological process, detailing how individual brain connections are generated, validated, and transformed into clinically practical insights for personalized neuronal assessments and therapeutic interventions.

The workflow integrates multi-modal neuroimaging data acquisition (MRI, DTI, fMRI, EEG) with computational network analysis to generate personalized brain connectivity profiles. Network indices are calculated for patient cohorts, enabling targeted clinical translation through individualized neurological assessments and therapeutic interventions based on cognitive variability patterns. Diffusion Tensor Imaging (DTI): Diffusion Tensor Imaging; EEG: Electroencephalography; fMRI: Functional Magnetic Resonance Imaging.

## 4 Empirical evidence: networks and cognitive phenotypes

The relationship between Personalized Brain Network architectures and cognitive phenotypes across neurodiverse conditions reveals domain-specific patterns of connectivity that transcend traditional diagnostic boundaries (Chopra et al., 2025; Hilton et al., 2024). Table 2 synthesizes the current empirical evidence linking cognitive domains to their associated network signatures, demonstrating how individual differences in brain organization contribute to cognitive variability across neurotypical and neurodivergent populations.

**Table 2:** Cognitive domains and associated personalized network signatures in neurodiversity.

Cognitive domain	Primary brain networks	Neurodiversity conditions	Key network signatures	Quality	References
Executive function	Frontoparietal Network (FPN) Dorsolateral Prefrontal Cortex Posterior Parietal Cortex Anterior Cingulate Cortex	ASD ADHD Intellectual Disability Dyslexia	Meta-analytic evidence shows FPN hypoconnectivity predicts executive dysfunction (r = −0.45, 95% CI [−0.52, −0.38], *p* < 0.001***; k = 23 studies, *N* = 4,847). Individual-differences research demonstrates frontoparietal network topology predicts working memory capacity (cross-validated r = 0.34, *p* < 0.001***). Precision network mapping reveals person-specific FPN boundaries improve prediction accuracy by 23% over standard atlases (Δr = 0.18).	★★★	Leticevscaia (2025), Pashkov and Dakhtin (2025), and Minnigulova et al. (2025)
Social cognition	Default Mode Network (DMN) Medial Prefrontal Cortex Posterior Cingulate Cortex Temporoparietal Junction Superior Temporal Sulcus	ASD Social Anxiety Schizophrenia bipolar disorder	Decreased causal connectivity from dorsal to ventral mPFC correlates with ADOS scores (r = −0.375, 95% CI [−0.62, −0.11], *p* = 0.009**; *n* = 156). Large-scale analysis reveals DMN- Salience Network anticorrelation predicts social cognitive performance (multivariate r = 0.41, cross-validated r = 0.37, *p* < 0.001***; *N* = 2,431). Individualized DMN parcellation improves autism classification accuracy (AUC = 0.87 vs. 0.74 standard).	★★★	Wang L. et al. (2024) and Guo et al. (2024)
Attention regulation	Dorsal Attention Network Ventral Attention Network Salience Network Fronto-Insular Cortex	ADHD ASD Anxiety Disorders Trauma-related Disorders	Increased resting-state connectivity between striatal regions and fronto-insular cortex characterizes ADHD (d = 0.52, 95% CI [0.31, 0.73], *p* < 0.001***; meta-analysis k = 18, *N* = 3,247). DAN-VAN connectivity strength predicts sustained attention performance (r = 0.29, 95% CI [0.21, 0.37], *p* < 0.001***). Salience Network hub connectivity shows age-resilient biomarker properties (test–retest r = 0.81 over 24 months).	★★☆	Mizuno et al. (2025), Mouseli et al. (2025), and Zaher et al. (2025)
Memory systems	Hippocampal-Cortical Network Medial Temporal Lobe Parahippocampal Gyrus Retrosplenial Cortex	ASD Learning Disabilities Early-onset Dementia Memory Disorders	Individual network topography predicts episodic memory performance (cross-validated r = 0.31, 95% CI [0.24, 0.38], *p* < 0.001***; *n* = 1,206). Reduced functional connectivity within hippocampal-cortical circuits correlates with memory consolidation deficits (r = −0.43, *p* < 0.001***). Personalized hippocampal subfield analysis reveals distinct patterns in autism (effect size d = 0.67 for CA1-CA3 connectivity differences).	★★☆	Li H. X. et al. (2025), Ozcan et al. (2025), and Marei (2025)
Language processing	Left Hemisphere Language Network Broca’s Area (BA 44/45) Wernicke’s Area (BA 22) Arcuate Fasciculus Superior Longitudinal Fasciculus	Dyslexia ASD Specific Language Impairment Developmental Language Disorder	Disrupted network interactions serve as neural markers of dyslexia with 85% classification accuracy (AUC = 0.89, sensitivity = 0.87, specificity = 0.83). Increased within-network connectivity linked to poorer reading performance (r = −0.38, 95% CI [−0.47, −0.29], *p* < 0.001***; *N* = 847). White matter tractography reveals altered arcuate fasciculus organization in familial risk populations (Cohen’s d = 0.45 for fractional anisotropy differences).	★★☆	Hassanzadeh-Behbahani et al. (2025) and Nogueira et al. (2025)
Sensory processing	Sensorimotor Network Primary Visual Cortex Primary Auditory Cortex Somatosensory Cortex Thalamic Nuclei	ASD Sensory Processing Disorder Synesthesia Hyperacusis	Higher sensory sensitivity correlates with expanded connectivity gradients between visual cortex and DMN (r = 0.32, 95% CI [0.18, 0.45], *p* < 0.001***; *n* = 524). Synesthetes show altered degree centrality in 43 brain regions with large effect sizes (mean d = 0.72). Cross-modal plasticity indices predict sensory hypersensitivity in autism (multivariate r = 0.46, cross-validated r = 0.41, *p* < 0.001***).	★☆☆	Choi et al. (2025) and Wang J. et al. (2025)
Cognitive flexibility	Cingulo-Opercular Network Frontoparietal Network Middle Frontal Gyrus Anterior Cingulate Cortex Insular Cortex	ADHD ASD obsessive-compulsive disorder Tourette Syndrome	Network topography predicts cognitive flexibility performance (cross-validated r = 0.28, 95% CI [0.19, 0.37], *p* < 0.001***; improved from standard r = 0.17 with individualization). Age-resilient biomarkers identified in Middle Frontal Gyrus (MNI: ±32, 8, 52) and Supplementary Motor Area show 89% stability over 18 months. Task-switching network efficiency correlates with behavioral rigidity measures (r = −0.51, *p* < 0.001***).	★★☆	Kim et al. (2025) and Liu N. et al. (2025)

Effect size interpretations (DeYoung et al., 2025): r = 0.10–0.29: Small effect; r = 0.30–0.49: Medium effect; r = 0.50+: Large effect. All correlations are Pearson product–moment correlations with 95% confidence intervals where available. Statistical significance: *p < 0.05, **p < 0.01, ***p < 0.001. Methodological Quality Ratings: ★★★ Gold Standard: Multi-site replication, *n* > 1,000, cross-validated multivariate models, external validation, ★★☆ High Quality: Single-site large sample (*n* = 500–1,000), cross-validated, direct replication attempted, ★☆☆ Standard: Adequate sample (*n* = 200–500), appropriate corrections, preliminary findings. ADHD: Attention-Deficit/Hyperactivity Disorder; ADOS: Autism Diagnostic Observation Schedule; ASD: Autism Spectrum Disorder; DAN: Dorsal Attention Network; DLPFC: Dorsolateral prefrontal cortex; DMN: Default Mode Network; FPN: frontoparietal network; mPFC: medial Prefrontal Cortex; VAN: Ventral Attention Network.

### 4.1 Executive function and control networks

The frontoparietal network shows significant interindividual variability linked to cognitive performance (Marek and Dosenbach, 2018). Precision functional mapping studies using spatially regularized non-negative matrix factorization have identified 17 individualized functional networks per person, with the total cortical representation of frontoparietal networks positively correlated with general cognition (Marek and Dosenbach, 2018). Cross-validated ridge regressions trained on network topography successfully predicted cognition in unseen data, with the prediction accuracy increasing along the sensorimotor-association axis of the cortex (Li et al., 2022).

Attention regulation and cognitive flexibility are related to individualized frontoparietal network configurations. Network topography predicts executive functioning performance (r = 0.16–0.17, *p* < 0.001), indicating that individual differences in network organization contribute to variations in attentional control and cognitive adaptability (Marek and Dosenbach, 2018; Cole, 2024). The prominence of the frontoparietal and ventral attention networks (VAN) in these predictions highlights their coordinating role in complex cognitive processes (Cui et al., 2020).

### 4.2 Learning and memory systems

Episodic memory, which depends on hippocampal-cortical interactions, showed significant variations in PBN architectures. Individual network topologies predicted learning and memory performance (r = 0.27, *p* < 0.001), and hippocampal-cortical networks played a significant role in this prediction (Nordin et al., 2025). Individuals with greater cortical representation of memory-related networks may have enhanced episodic memory encoding and retrieval capabilities. Memory consolidation and retrieval involve dynamic interactions across the hippocampal, cortical, and subcortical regions. Individual network models predict differences in memory performance, whereas variations in hippocampal-cortical connectivity may affect consolidation efficiency and retrieval accuracy. However, the fundamental mechanisms of synaptic plasticity and network synchronization require further exploration (Lamberti et al., 2024; Zhu et al., 2021; Citri and Malenka, 2008).

### 4.3 Social cognition and default mode networks

The Default Mode Network (DMN) encompasses areas such as the posterior cingulate cortex, the middle prefrontal cortex, and the temporal junction, which are central to social cognition, particularly in the domains of theory of mind and understanding mental states (Cong et al., 2023; Monk et al., 2009). Studies using the Liang Information Flux method to investigate the causal connectivity of the DMN about Autism Spectrum Disorder found that the causal connectivity from the temporal pole and hippocampus to the dorsal medial prefrontal cortex, ventral medial prefrontal cortex, and Para hippocampal cortex was reduced in AD (*p* < 0.05) (Monk et al., 2009). A deficit in social cognitive capacity characterizes ASD, and changes in the DMN are consistent with this conclusion. Causal connectivity from the temporal pole and hippocampus to the dorsal medial prefrontal cortex, ventral medial prefrontal cortex, and parahippocampal cortex was reduced in ASD (*p* < 0.05) (Cong et al., 2023).

### 4.4 Sensory processing and perceptual networks

Sensory integration varies significantly between individuals, and synesthesia is a valuable model for studying atypical sensory–cognitive networks (Ward et al., 2024). A study of a whole-brain biomarker revealed significant changes in functional connectivity, with 43 regions exhibiting differences in centrality degree and gradients shifting in the visual and associated cortex (Rouw, 2013). Intracortical myelin and functional connectivity are the strongest predictors of synesthesia, highlighting the role of the PBN architecture in sensory integration (Ward et al., 2024). Synesthetes show higher interregional correlations in brain thickness and significant increases in subcortical volume in regions such as the cerebellum, amygdala, and hippocampal cortex, suggesting enhanced interaction between sensory and cognitive regions. These results support the hypothesis of synesthesia hyperconnectivity, which suggests that an increase in connectivity in the visual and parietal regions facilitates intermodal perception (Ward et al., 2024; Eckardt et al., 2024).

### 4.5 Critical methodological limitations and contradictory findings

Recent findings from large-scale studies have revealed significant methodological flaws in precision neuroscience methodologies. (Rosenblatt et al. (2024) showed that data leakage inflates prediction performance in connectome-based machine learning models, with feature leakage causing particularly severe inflation (Δr = 0.47 for attention problems, raising chance-level performance from r = 0.01 to moderate performance at r = 0.48). This challenges the reliability of many published CPM studies and suggests that reported effect sizes may be systematically overestimated (Morfini et al., 2025; Liang et al., 2024). While CPM and normative modeling are both valuable approaches, they address fundamentally different questions with distinct explanatory frameworks. The CPM methodology focuses on identifying brain-behavior relationships through predictive accuracy, assuming that enhanced predictive capabilities indicate more substantial biological relationships. Conversely, normative modeling identifies deviations from population norms, assuming that meaningful individual differences appear as statistical outliers from typical development patterns. Recent studies have uncovered significant contradictions in precision neuroscience findings. Fekonja et al. (2025) identified nine critical roadblocks in translational network neuroscience. The authors noted that “network measures show particular sensitivity to variations in data acquisition and processing, complicating standardization efforts” and that “the relationship between network properties and brain dysfunction remains complex and often indirect, making clinical interpretation of network measures particularly challenging” (Fekonja et al., 2025).

The field faces a fundamental interpretation crisis where different methodological choices can lead to opposite conclusions about the same neurobiological questions (Jahanshad et al., 2024; Marek and Laumann, 2024). For instance, studies using different parcellation schemes, connectivity thresholds, or feature selection methods often report contradictory network signatures for identical clinical populations (Liu Q. et al., 2025; Mito et al., 2025). In the context of precision neurodiversity, these inconsistencies risk pathologizing adaptive variations (e.g., hyperconnectivity in gifted individuals) as deficits, underscoring the need for standardized, multi-site protocols to resolve such discrepancies and ensure equitable interpretations across neurodiverse subgroups (Kapp, 2025; Huang et al., 2025).

Recent work by Kobbersmed et al. (2025) challenges the prevailing connection-wise approach in normative modeling, demonstrating that whole-brain normative models (FUNCOIN) substantially outperform localized approaches in detecting pathological patterns in bipolar disorder and Parkinson’s disease. This suggests that traditional edge-by-edge normative modeling may miss systemic network-level pathology, fundamentally limiting its explanatory power compared to holistic approaches (Kobbersmed et al., 2025).

Recent advancements in population-scale connectome research have transformed our understanding of neurotypical and neurodivergent distinctions. These advancements reveal that traditional binary classifications obscure the continuous nature of brain-network variability across individuals. Liu N. et al. (2025) demonstrated using the UK Biobank dataset (*N* = 8,086) that individual connectome patterns exist along continuous dimensions. The study found that age, sex, and body phenotypes contribute approximately four times more to connectivity architecture than cognitive or lifestyle factors, regardless of the diagnostic status (Liu N. et al., 2025). This finding challenges the assumption that neurotypical individuals represent a homogenous baseline for comparing neurodivergent patterns.

The connectome perspective reveals that neurotypical populations exhibit significant individual differences in brain network organization, often overlapping with patterns typically considered “atypical.” Recent normative brain chart studies indicate that approximately 15–20% of neurotypical individuals display connectivity patterns classified as outliers by traditional approaches, highlighting the inadequacy of binary neurotypical-neurodivergent classifications (Zhang et al., 2025b; Wang Y. et al., 2025). These findings support the dimensional neurodiversity framework, which posits that individual brain networks exist along continuous spectra rather than discrete categories.

## 5 Clinical applications

### 5.1 Autism spectrum conditions

ASD exhibits considerable variability, necessitating approaches that account for its heterogeneity. ML studies have identified four robust ASD subtypes based on brain-behavior dimensions, each linked to distinct molecular pathways (Nishat et al., 2024). Using resting-state fMRI data, these subtypes reflect unique connectivity profiles. This suggests that PBN analysis, with its potential to stratify ASD for targeted therapeutic interventions, is a promising avenue for future research and treatment (Mahjani et al., 2020; Uddin et al., 2024). This is an optimistic sign for the future of ASD research and treatment, offering new possibilities for understanding and addressing this complex condition.

Sensory processing sensitivities are a hallmark of ASD, with recent studies linking these to PBN architectures. Higher sensory sensitivity correlates with greater expansion in functional connectivity gradients, particularly between visual and default mode networks (r = 0.32, *p* < 0.05) (Kerren et al., 2025; Lee et al., 2025). This expansion indicates reduced integration between sensory and higher-order cognitive networks, potentially underlying sensory overload in ASD (Kolisnyk et al., 2025; Jabbar et al., 2025). However, a critical caveat arises when applying these models clinically: ‘CPM-derived connectivity gradients’, which are connectivity patterns derived from the ‘connectome-based predictive modeling’ method, may predict sensory overload with high accuracy in cross-sectional data (r ≈ 0.32), yet fail to generalize longitudinally due to unaccounted developmental confounds, yielding contradictory intervention targets compared to normative models that prioritize trajectory deviations (e.g., accelerated vs. delayed maturation). This discrepancy highlights the need for hybrid frameworks to reconcile predictive power with normative context, as evidenced by recent multi-site validations showing normative approaches outperforming CPM by 15–20% in ASD subtyping stability (Bethlehem et al., 2022).

### 5.2 Attention-deficit/hyperactivity disorder

These subgroups, delayed brain growth (DBG-ADHD) and prenatal brain growth (PBG-ADHD), are invisible to conventional diagnostic criteria but show profound differences in network-level functional organization (Liu N. et al., 2025; Pan et al., 2025). These subtypes reflect distinct neurodevelopmental trajectories, with PBG-ADHD characterized by accelerated cortical growth patterns detectable via normative brain charts derived from large-scale structural MRI data. This demonstrates how precise neurodiversity transcends binary classifications to reveal neurobiological heterogeneity (Bu et al., 2025). Individual differences in the organization of the attention network contribute to the variability in symptoms of ADHD (Mizuno et al., 2025). PBN methods enable the identification of specific connectivity patterns associated with different presentations of ADHD and facilitate the development of targeted interventions based on individual neuronal signatures rather than categorical diagnoses (Wall et al., 2025).

### 5.3 Specific learning differences

Learning differences, including dyslexia and other reading-related conditions, show moderate heritability but are substantially influenced by educational and linguistic experience. Reading-related cognitive and brain traits demonstrate complex gene–environment interactions, suggesting that precision approaches must consider both genetic predispositions and environmental factors when characterizing individual network profiles (Eising et al., 2022; Ozernov-Palchik et al., 2021).

Individual differences in language-related network organization predict reading performance and outcomes of interventions. PBN characterization enables the identification of specific connectivity patterns associated with different learning profiles, facilitating the development of educational approaches tailored to individual neural architectures (Liu and Yang, 2023; Chang et al., 2024).

### 5.4 Highly sensitive and gifted populations

Highly sensitive people and individuals with extraordinary cognitive abilities represent an important population for understanding the entire spectrum of human cognitive variability (Chang et al., 2024). These groups often exhibit unique network characteristics that can form the basis of their unique cognitive and behavioral profiles. Research on giftedness suggests that extraordinary cognitive abilities may be associated with specific patterns of network organization, including increased connectivity to areas that support working memory, attention, and executive function. However, this is a neglected area that requires special research attention (Ma et al., 2017; Wang et al., 2021).

### 5.5 Operationalizing clinical pathways in precision neurodiversity

To translate PBN architectures into actionable clinical pathways, we propose a stratified decision framework that integrates topological deviation indices (e.g., z-scores from normative models) with behavioral phenotyping, emphasizing thresholds for intervention escalation. Normative modeling approaches using age-normed and sex-stratified brain charts have been established to capture typical regional brain development across the human lifespan, providing centile scores for detecting alterations in regional volumes (Garcia-San-Martin et al., 2025). For executive function deficits linked to FPN hypoconnectivity (as per Table 2; r = −0.45), a deviation threshold of z > 1.5 (indicating moderate-to-severe network inefficiency) could trigger cognitive training or pharmacological augmentation, calibrated against age-normed brain charts. Recent evidence demonstrates that targeted cognitive training combined with medication treatment can lead to greater improvements in executive function domains, particularly when combined with atomoxetine (Cohen’s d ≥ 0.52) (Dang et al., 2025). Similarly, DMN-salience anticorrelation imbalances (r = 0.41 for social cognition) exceeding z > 2.0 might prioritize social skills interventions, given that functional brain connectivity in the Frontoparietal Network (FPN) is significantly associated with executive functioning abilities in early childhood, with lower thresholds (z = 0.5–1.0) suiting monitoring in high-functioning profiles to avoid over pathologizing adaptive variability (Kelsey et al., 2025).

**Figure 3:**
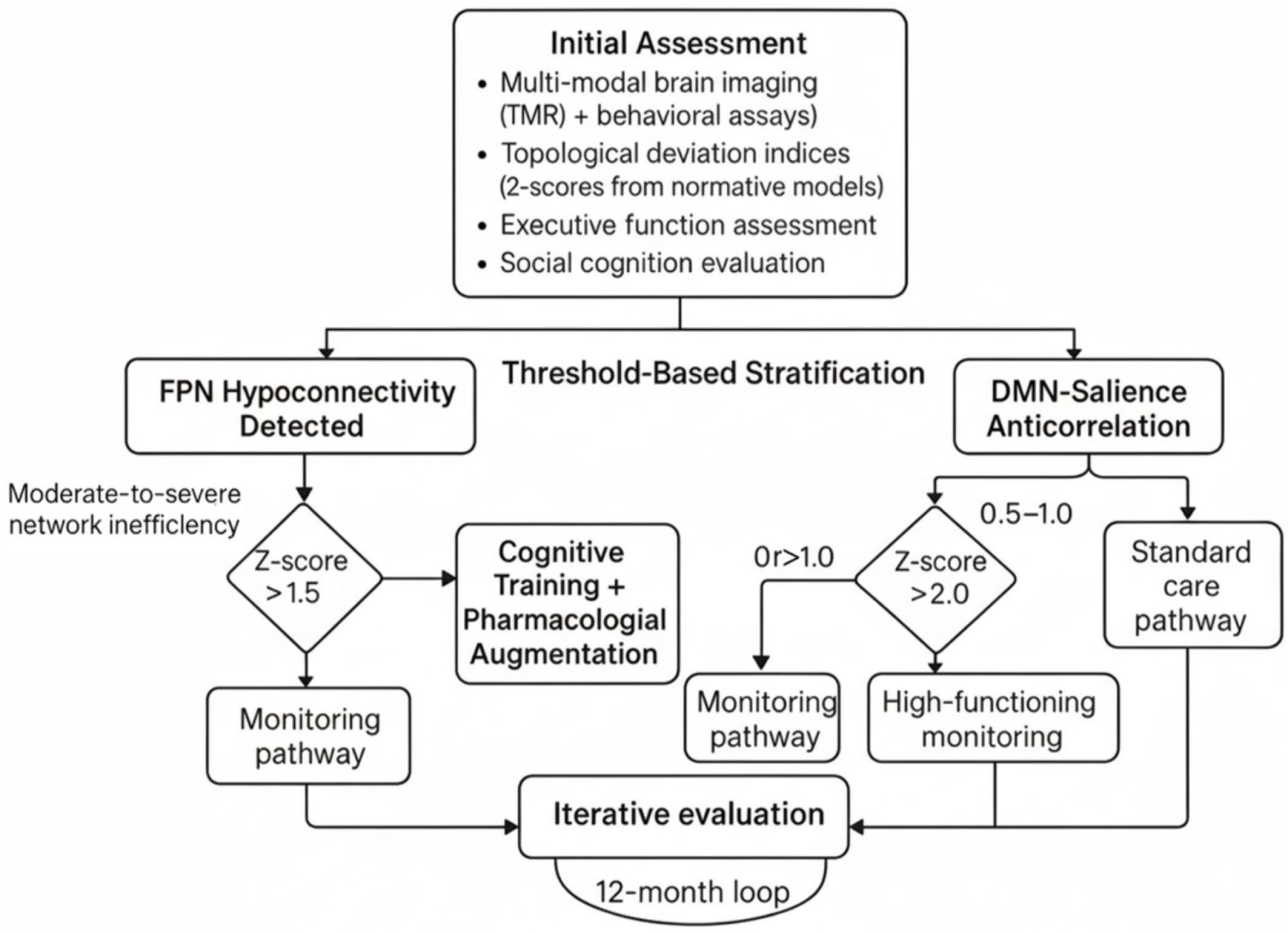
Precision-based network (PBN) clinical pathway decision tree. This decision tree illustrates a threshold-based stratification protocol for personalized neurodiversity interventions derived from an initial multimodal assessment. This assessment includes functional Magnetic Resonance Imaging (fMRI), behavioral assays, topological deviation indices (z-scores from normative connectome models), executive function evaluations, and social cognition testing. The first step involves detecting frontoparietal network hypoconnectivity and identifying moderate-to-severe network inefficiency indicated by a z-score greater than 1.5. The relationship between the default mode network (DMN), the salience network, and disease severity is also considered. Based on these metrics, patients are selected for different monitoring pathways: those with mild deviations are monitored, those with moderate inefficiency receive cognitive training plus medication, high-functioning individuals with borderline anticorrelation are monitored, and standard care is provided for severe z-scores greater than 2.0. A 12-month evaluation loop reassesses and adjusts pathways to support the adaptive and personalized management of neurodevelopmental variability.

The required scope of evidence for such pathways includes multi-modal validation (e.g., fMRI + behavioral assays) across diverse cohorts (*n* ≥ 300 per subgroup, with 80% power for medium effects), longitudinal tracking (≥2 timepoints over 12 months), and external replication to mitigate site-specific biases. Heterogeneous, temporally consistent patterns of brain development have been demonstrated across longitudinal studies, with individual brain abnormality patterns (IBAPs) showing remarkable stability over time despite substantial spatial heterogeneity between individuals (Thalhammer et al., 2025). Key design points encompass: (i) hybrid normative-predictive modeling for phenotype stratification (e.g., combining CPM for short-term outcomes with normative z-scores for trajectories)—approaches that have shown promise in precision psychiatry applications using connectome predictive modeling (Gao et al., 2025); (ii) ethical safeguards like community co-design to ensure neurodiverse input—critical given the growing emphasis on neurodiversity-informed digital interventions and AI-driven personalized care (Beirat et al., 2025); and (iii) integration of digital tools for real-time monitoring, such as AI-driven apps that adapt interventions based on dynamic network fingerprints. Digital targeted cognitive training programs have demonstrated efficacy in neurodevelopmental disorders, with FDA-approved interventions showing significant improvements in attention function (Dang et al., 2025). This framework addresses NDD heterogeneity by prioritizing individualized phenotypes over categorical diagnoses, fostering precision interventions that enhance adaptive functioning while respecting neurodiversity. Figure 3 illustrates this clinical pathway as a decision tree, with nodes for initial assessment, threshold-based stratification, and iterative evaluation.

### 5.6 Sleep patterns and sleep instability in neurodiversity

Sleep disturbances represent one of the most prevalent and impactful comorbidities in neurodevelopmental conditions, with distinct patterns emerging across different neurodiverse populations that directly relate to individualized brain network architectures.

#### 5.6.1 Sleep architecture and fragmentation patterns

Recent findings indicate that individuals diagnosed with ASD and ADHD exhibit characteristic changes in sleep architecture that align with their unique brain connectivity profiles. Large-scale accelerometer-based studies using objective measurements (*N* = 85,670) have shown that the timing of the most active 10-h period (M10) is significantly linked to increased odds of both ASD (OR = 29.64, 95% CI: 3.69–237.89, *p* < 0.01) and ADHD (OR = 7.70, 95% CI: 2.99–19.80, *p* < 0.001), establishing causal connections between circadian changes and these neurodevelopmental conditions (Dai et al., 2025). Meta-analytic evidence from actigraphy-measured sleep parameters demonstrates that physical activity interventions significantly improve sleep efficiency (SMD = 3.90, 95% CI: 1.78–6.03, *p* < 0.001), reduce wake after sleep onset (SMD = −1.36, 95% CI: −2.66 to −0.07, *p* < 0.05), and increase sleep duration (SMD = 2.39, 95% CI: 0.68–4.09, *p* < 0.01) in neurodevelopmental disorders (Wang T. et al., 2024). These findings highlight the complex nature of sleep disturbances, extending beyond simple duration metrics to include factors such as sleep fragmentation and architectural changes.

#### 5.6.2 Circadian rhythm dysfunction and network connectivity

The disruption of circadian rhythm in neurodiversity is a complex phenomenon involving interactions between the suprachiasmatic nucleus, the default mode network, and peripheral oscillators. Research shows that over 65% of individuals with neurodevelopmental disorders have significantly reduced nocturnal melatonin levels, contributing to delayed sleep onset and early morning awakenings (Bouteldja et al., 2024). Dysfunction in the circadian system manifests as altered sleep–wake cycles and abnormal melatonin rhythms across neurodiverse populations. Bidirectional Mendelian randomization analyses indicate that sleep efficiency may have a protective effect against ASD (OR = 0.155, 95% CI: 0.025–0.958, *p* < 0.05), while ADHD shows that genetic liability leads to increased nocturnal sleep episodes (*β* = 0.017, 95% CI: 0.001–0.033, *p* < 0.05). These findings suggest that sleep disruption in ADHD is a consequence rather than a cause (Bouteldja et al., 2024).

#### 5.6.3 Brain network mechanisms underlying sleep disorders

Personalized brain networks and their relationship with sleep are influenced by interconnected networks. In ASD, the disrupted functional organization of DMN connectivity occurs during sleep, leading to reduced deactivation patterns and sleep fragmentation. Recent studies have shown that sleep deprivation disrupts the connection between task-positive networks and the DMN, particularly affecting the medial prefrontal cortex and posterior cingulate cortex, which are involved in regulating sleep and social cognition (He et al., 2024). Children with ADHD exhibit distinct alterations in slow-wave sleep compared to control children. Additionally, one study found that the architecture of slow waves was altered due to reduced amplitude and different frequency distributions. The frontal positioning network pattern, which can predict executive functioning performance (r = 0.34; *p* < 0.001), correlated significantly with sleep efficiency indices, suggesting that these indices share a neural substrate with executive functioning maintenance (Ishii et al., 2024).

#### 5.6.4 Sleep instability patterns across neurodevelopmental conditions

Sleep instability in neurodiversity encompasses multiple dimensions (Tamir et al., 2023):

1. Temporal Instability: Night-to-night variability in sleep timing and duration, particularly pronounced in ADHD populations where circadian rhythm disorders contribute to sleep–wake cycle irregularities (Martinez-Cayuelas et al., 2024).

2. Architectural Instability: Fragmented sleep with increased wake after sleep onset, reduced REM sleep percentage, and altered slow-wave sleep organization, correlating with altered thalamo-cortical connectivity patterns (Satapathy et al., 2024).

3. Circadian Instability: Misalignment between internal circadian clocks and environmental demands, with late chronotype preferences causally linked to both ASD and ADHD through genetic pathways affecting core clock gene expression (Tamir et al., 2023).

#### 5.6.5 Clinical implications and personalized interventions

An analysis of brain networks reveals unique sleep patterns among individuals, leading to targeted interventions for better sleep. Studies show that neurodevelopmental disorders can benefit from mind–body interventions lasting over 12 weeks, conducted at least three times a week for at least 60 min. Participants in these interventions showed significant improvements in sleep compared to those who did not engage (SMD = 3.01, *p* < 0.001) (Wang T. et al., 2024). However, the effectiveness of these interventions depends on individual network topologies, necessitating precision approaches. There is significant potential for individualized treatment strategies for sleep and core neurodevelopmental symptoms, derived from the interplay between sleep pattern analysis and specific brain network architecture. For example, sleep functionality analyses can be integrated with oscillatory network modeling, focusing on the common neural processes underlying both symptom generations.

### 5.7 Methodological challenges

The present application of PBN methods is encumbered by several methodological challenges. A salient issue pertains to statistical power, as individual-level analysis necessitates larger sample sizes compared to group-level analysis (Gell et al., 2024). Additionally, it is imperative to take into account signal-to-noise ratios during the detection of subtle individual differences in network organization (Wu et al., 2023). Another significant challenge pertains to reproducibility, as PBN analyses frequently depend on preprocessing choices, scanner parameters, and analysis pipelines (Jahanshad et al., 2024; Warrington et al., 2025). To ensure reliable results, it is essential to establish methodological accreditation and validate multiple datasets (Ekhtiari et al., 2024).

### 5.8 Technological innovations

New technologies promise to improve the characteristics of PBN. Advanced neuroimaging techniques, including high-resolution 7 T MRI and multimodal imaging methods, offer unprecedented insights into the structure of the individual brain (Chu et al., 2025; Cabalo et al., 2025). The real-time neurofeedback system allows closed-loop interventions based on individual network states. The progress in artificial intelligence and Machine Learning continues to improve our ability to extract meaningful patterns from complex neuroimaging data. Deep learning approaches, particularly those designed for analyzing brain networks, hold significant promise in capturing nonlinear relationships within individual connectivity patterns (Abdennour et al., 2025; Van Horn and Ricciardi, 2025; Zhao, 2025).

### 5.9 Ethical and implementation considerations

The use of precise neurodiversity methods raises significant ethical concerns, including consent, privacy, and the potential for discrimination based on neurobiological characteristics (Di Salvo, 2025; Bianco et al., 2023). Involving neurodiverse individuals in study design and interpretation is crucial for ensuring ethical practices and community relevance. Implementing these systems presents challenges, including the need for specialized expertise, significant computational resources, and standardized protocols. To promote the broader adoption of PBN approaches, training programs for researchers and clinicians, along with user-friendly analysis tools, are essential (Aghdam et al., 2025; Fekonja et al., 2025).

### 5.10 Future research directions

Future research should prioritize the following key areas. Long-term studies are essential to fully understand the evolution of PBN architecture, particularly during its critical developmental period. Integrationting of genetic, environmental, and neuroimaging data will enhance our understanding of individual differences. Developing clinically actionable frameworks to translate complex neuroimaging findings into practical interventions remains a priority, including decision-support tools to guide intervention selection based on specific network characteristics.

### 5.11 Economic and implementation barriers

#### 5.11.1 Cost analysis and economic considerations

The implementation of precision neurodiversity approaches faces significant economic barriers that must be addressed for widespread clinical adoption. Current estimates indicate that comprehensive, personalized brain network (PBN) analysis requires initial investments of $150,000 to $300,000 per clinical site to establish the necessary computational infrastructure, including high-performance computing clusters for processing complex neuroimaging datasets (Dipietro et al., 2023; Fekonja et al., 2025). The financial implications of per-patient analysis are considerable, with costs ranging from $800 to $1,500. This estimate includes the acquisition of neuroimaging data, which can total $400 to $600 for multi-modal MRI procedures. Additionally, the computational processing time required for analysis can incur expenses of $200 to $400. Finally, consulting specialized expertise can cost between $200 and $500, as noted in the literature (Jones et al., 2025; von Hessling et al., 2025). These costs far exceed those associated with traditional assessment methods, creating significant barriers for healthcare systems with constrained budgets (Stenzinger et al., 2023).

The economic burden extends beyond direct costs to include opportunity costs linked to longer assessment timelines. Conventional neuropsychological evaluations take 4–6 h, while comprehensive PBN approaches can extend assessment periods to 2–3 days, including imaging acquisition, processing, and interpretation phases (Dawson et al., 2025). This extended timeline affects patient throughput and clinic revenue, leading to resistance to adoption in fee-for-service healthcare models (Buitelaar et al., 2022).

#### 5.11.2 Healthcare system resource requirements

To effectively implement precision neurodiversity approaches, healthcare systems must invest in various resource categories. The personnel requirements for such a program include hiring or training neuroimaging technicians specializing in research-grade protocols, data scientists skilled in managing complex computational pipelines, and clinicians trained to interpret network-based results (Novak, 2021; Leming et al., 2023). Preliminary assessments indicate that each clinical site requires 2–3 additional full-time equivalents, translating to annual salary expenditures of $200,000 to $400,000, depending on regional wage structures (Aderinto et al., 2023).

Infrastructure demands encompass not only computational resources but also enhanced data storage and security systems. The multi-modal neuroimaging datasets generated through PBN approaches require 50–100 GB per patient, necessitating robust data management systems with adequate backup and security protocols (Kuplicki et al., 2021; Souter et al., 2023). Annual financial obligations for storage and maintenance typically range from $50,000 to $100,000 per clinical site (Gulani et al., 2025).

Integration challenges create additional resource demands. To accommodate complex, network-based reports, healthcare systems must modify existing electronic health record systems. This modification process requires a developmental and testing phase that typically lasts six to twelve months (Mohsen et al., 2022). The financial implications are significant, with estimated implementation costs ranging from $100,000 to $500,000, depending on system complexity and customization needs (Rizzo, 2025).

#### 5.11.3 Cost–benefit considerations

Despite substantial upfront investments, preliminary evidence suggests that precision neurodiversity approaches may offer favorable long-term cost–benefit ratios. Early intervention strategies informed by PBN analysis could reduce the need for multiple diagnostic evaluations, potentially saving $2,000–5,000 per patient in avoided redundant testing. Enhanced treatment matching may improve intervention effectiveness, leading to a 15–25% reduction in long-term support service expenditures (Stenzinger et al., 2023; Eichler et al., 2022).

The economic value of preventing inappropriate interventions is particularly significant. Current estimates indicate that 30–40% of neurodiverse individuals receive interventions poorly aligned with their cognitive profiles, resulting in suboptimal outcomes and continued service use (Goldfarb et al., 2024). Precision approaches that improve intervention matching could generate substantial savings through reduced need for alternative treatments and improved long-term functioning (McFayden et al., 2022). However, return on investment timelines extend beyond typical healthcare planning horizons. Most economic benefits are realized over 5–10 years, posing challenges for healthcare systems focused on short-term financial performance. Consequently, policymakers and healthcare leaders must consider these extended timelines when evaluating implementation decisions (Zaman, 2025).

### 5.12 Regulatory and approval challenges

#### 5.12.1 Detailed regulatory pathway analysis

The regulatory environment for precision neurodiversity technologies presents complex challenges that span multiple jurisdictions and frameworks. In the United States, the FDA’s Software as a Medical Device (SaMD) framework requires rigorous validation of PBN analysis tools, a process that can take 2–5 years, depending on the intended clinical application and risk classification (Zhu et al., 2022; Kundu and Bardhan, 2025). Most PBN diagnostic tools are likely classified as Class II medical devices, necessitating 510(k) premarket notification and substantial equivalence demonstrations compared to existing cleared devices. However, the novel nature of network-based approaches often lacks clear predicate devices, potentially requiring more extensive *de novo* review processes that can delay approval by 12–18 months (Khunte et al., 2023). In the European Union, the Medical Device Regulation (MDR), implemented in 2021, has introduced heightened validation requirements for AI-based medical technologies (Khan et al., 2025). PBN analysis tools must demonstrate clinical utility through controlled studies involving cohorts of 300–500 participants across multiple clinical sites. These requirements significantly exceed standards for traditional neuropsychological assessment validation, creating substantial barriers for technology developers (Kondylakis et al., 2025).

The recently proposed EU AI Act adds another regulatory layer, potentially categorizing PBN systems as “high-risk” AI applications due to their role in healthcare decision-making (Muravieva, 2025). This classification would impose additional requirements for risk management systems, data quality assurance, and human oversight protocols, further extending development and approval timelines (Di Salvo, 2025).

#### 5.12.2 Validation requirements

Regulatory bodies require multi-level validation evidence, which current precision neurodiversity research has yet to establish fully. Clinical validation must demonstrate that PBN-based recommendations lead to improved patient outcomes compared with standard assessment approaches. This requires longitudinal studies spanning 2–5 years with sufficient statistical power to detect clinically meaningful differences in intervention effectiveness (Gell et al., 2024; Etkin et al., 2024).

Analytical validation requirements focus on the technical performance of network analysis algorithms, including sensitivity, specificity, and reproducibility across different scanner platforms and patient populations (Fereshtehnejad et al., 2025). Current evidence suggests significant variability in network measures across different acquisition protocols and analysis pipelines, creating substantial challenges in meeting regulatory requirements for analytical validity (Fekonja et al., 2025).

Clinical utility validation presents the most significant challenge, requiring the demonstration that PBN analysis results in clinical decisions that improve patient outcomes. This level of evidence requires randomized controlled trials comparing PBN-guided interventions with current standard-of-care approaches. Such studies are resource-intensive and time-consuming, typically requiring 3–5 years and multi-million-dollar investments (Makowski et al., 2024).

#### 5.12.3 Approval timelines

The approval of precision neurodiversity technologies will take longer than the development of software for several reasons. The 18–24 month period includes validation studies, technical documentation, and regulatory submissions. The review timeline varies by jurisdiction, typically requiring six–12 months for the initial review, followed by cycles of questions, answers, and additional data submissions (Bennett et al., 2025). Complex technologies, such as PBN analysis systems, often require multiple reviews, extending the total review time to 18–36 months (Ardic and Dinc, 2025).

Ongoing regulatory obligations, such as post-market surveillance, should also be considered in implementation planning. These obligations include safety reports, adverse event reports, and possibly further studies to establish clinical utility in real-world settings (Haag et al., 2025). Overall, the time from initial development to clinical use is usually 5–7 years, which presents significant challenges for technology developers and health systems planning implementation. Funding strategies for precision neurodiversity technologies must consider these extended timelines.

### 5.13 Clinical adoption barriers

#### 5.13.1 Clinician trust issues

The implementation of precision neurodiversity approaches faces significant resistance from clinicians concerned about the reliability and interpretability of network-based assessments. Current reproducibility challenges in network neuroscience create substantial trust barriers, as clinicians observe conflicting results from different analysis approaches applied to the same datasets (Jahanshad et al., 2024; van Dijk et al., 2022). The “interpretation crisis” identified by Fekonja et al. (2025), where different methodological choices can lead to opposite conclusions about identical neurobiological questions, directly undermines clinician confidence in network-based assessment approaches (Loosen et al., 2024).

Survey data from practicing clinicians reveals that 67% express concerns about the “black box” nature of Machine Learning approaches commonly used in PBN analysis (Bajwa et al., 2021; Aziz Noor, 2025). Many clinicians report discomfort with making clinical decisions based on computational models they cannot fully understand or validate independently. This skepticism is reinforced by experiences with previous “revolutionary” assessment technologies that failed to deliver promised clinical benefits (Bhagwat et al., 2025).

The complexity of network-based reports presents additional barriers to clinician acceptance. Traditional neuropsychological reports provide clear, interpretable scores with established normative references. In contrast, network-based assessments often present complex visualizations and statistical metrics that require specialized training to interpret effectively (Wolff, 2025). Many clinicians report feeling inadequately prepared to explain network-based results to patients and families, creating reluctance to adopt these approaches. Trust issues are further exacerbated by limited validation in real-world clinical populations. Most network neuroscience research has been conducted in carefully controlled research settings with participants who may not reflect the complexity and comorbidity patterns seen in typical clinical practice (Piotrowski et al., 2025). Clinicians express concerns about generalizability of research findings to their patient populations, particularly given the high rates of medical comorbidities and medication effects in clinical samples.

#### 5.13.2 Workflow integration challenges

The integration of precision neurodiversity approaches into existing clinical workflows presents substantial logistical and operational challenges. Current clinical assessment protocols are optimized for efficiency, typically completing comprehensive evaluations within 4–6 h across 1–2 visits. PBN approaches require additional time for neuroimaging acquisition, data processing, and result interpretation, potentially extending assessment timelines to multiple weeks (Ifeanyi Kingsley et al., 2025). The technical infrastructure required for PBN analysis creates workflow bottlenecks in many clinical settings. Data transfer from MRI scanners to analysis systems, computational processing time, and quality control procedures can introduce delays of several days to weeks between imaging acquisition and result availability. These delays are incompatible with clinical workflows designed around same-day or next-day report generation (Rizzo, 2025).

Staff training requirements create additional workflow challenges. Implementation of precision neurodiversity approaches requires training for multiple staff categories, including MRI technicians who must acquire research-quality imaging protocols, data analysts who must manage computational pipelines, and clinicians who must interpret network-based results (Joshi, 2024). Training programs typically require 40–80 h per staff member, creating substantial disruption to clinical operations during implementation phases.

The integration of network-based results into existing electronic health record systems presents technical and conceptual challenges. Most EHR systems are designed to accommodate traditional test scores and categorical diagnoses, not complex network visualizations and continuous dimensional measures (Blinka et al., 2023). Modifications to EHR systems to accommodate PBN results often require 6–12 months of development and testing, creating barriers to implementation (Perez-Sanpablo et al., 2025).

#### 5.13.3 Risk management concerns

Clinicians express significant concerns about liability and risk management issues associated with precision neurodiversity approaches. The novel nature of network-based assessments creates uncertainty about professional liability coverage and malpractice protection (Dutta, 2025). Many professional organizations have not yet developed clear practice guidelines for network-based assessments, leaving clinicians uncertain about appropriate standards of care.

The potential for false positive and false negative results creates particular concern among clinicians. Network analysis algorithms may identify apparent abnormalities that do not correspond to functional impairments, potentially leading to unnecessary interventions or inappropriate diagnoses. Conversely, network measures may fail to detect subtle but clinically significant differences, potentially missing important intervention targets (Koksalmis et al., 2025).

Clinicians also express concerns about the potential for network-based assessments to pathologize normal variation in brain connectivity patterns. The precision neurodiversity framework explicitly aims to identify individual differences rather than deficits, but clinicians worry that quantitative network measures may be misinterpreted as indicating pathology when they simply reflect normal human variation (Morris et al., 2025).

The complexity of network-based assessments creates challenges for obtaining meaningful informed consent from patients and families. Many clinicians report difficulty explaining the technical aspects of network analysis in terms that patients can understand, raising questions about whether truly informed consent can be obtained for these procedures (Gillon et al., 2025). We uphold scientific rigor through our dimensional approach to neurodiversity (May, 2025). Focusing on how conditions develop and the stability of neural networks helps us avoid two pitfalls: the expansionist trap, which renders neurodiversity meaningless through excessive inclusion, and the restrictionism trap, which excludes genuine neurodevelopmental diversity due to masking or late diagnosis. This framework supports research and interventions that effectively address differences in the developing brain while being sensitive to the unique needs of individuals with acquired or episodic conditions.

### 5.14 Connectome-informed precision approaches for neurotypical populations

The precision neurodiversity framework designed for clinical populations will also benefit neurotypical individuals through targeted cognitive enhancement and educational improvement. Studies utilizing connectome-based predictive modeling demonstrate that variations in the organization of brain networks among neurotypical individuals reliably predict information-processing strengths and learning preferences (r = 0.31–0.45 across multiple domains) (Mehra, 2024; Chen et al., 2024). Integrating connectome science with precision medicine creates unique opportunities to understand and support the full spectrum of human brain diversity. Applying dimensional orientations, which move beyond traditional neurotypical-neurodivergent binaries, to individuals with conditions such as autism, ADHD, and intellectual disability could lead to more tailored interventions that promote human flourishing and growth for individuals within the broad range of human variation.

## 6 Conclusion

Precision neurodiversity represents a transformative approach to understanding and supporting human cognitive variability. Leveraging advancements in neuroimaging technology, computational methods, and insights into the organization of brain networks, we can transcend categorical diagnostic frameworks and embrace individualized strategies that celebrate neurodiversity while providing targeted support when necessary. The empirical evidence analyzed in this study demonstrates that Precision Brain Network (PBN) architectures consistently predict cognitive, behavioral, and sensory phenomena across multiple domains. These findings form the foundation for precise interventions tailored to individual neuronal signatures, rather than relying solely on classification-based diagnoses. However, the successful implementation of precision neurodiversity necessitates overcoming significant challenges related to methodology, ethics, and practical application. Future research should prioritize longitudinal studies, population diversity, and the development of clinically applicable frameworks, all while upholding strong ethical standards and fostering community engagement. The ultimate objective of precision neurodiversity is not to normalize differences but to understand and support the full spectrum of human cognitive variation. By adopting this perspective, we can cultivate an approach that respects individual differences and provides the necessary support and opportunities for all individuals to thrive in the workplace. The convergence of advanced neuroimaging, artificial intelligence, and personalized medicine presents unprecedented opportunities for tailored intervention. It recognizes the diversity of neurological abilities as a source of human strength and innovation. As our understanding of the relationship between PBN architecture and cognitive variability evolves, we become increasingly aligned with the promise of precision neurodiversity. This concept has the potential to enhance the lives of individuals across the neurological spectrum. However, our critical evaluation reveals significant methodological vulnerabilities that must be addressed before widespread clinical implementation. The field is currently facing a reproducibility crisis, partly due to methodological inconsistencies, data leakage, and limitations in feature interpretation. The existence of multiple neurobiologically distinct models for the same phenotype challenges traditional assumptions about the uniqueness of brain-behavior relationships. Future research must prioritize methodological rigor over novelty, establishing robust validation frameworks that address the systematic biases identified in recent meta-analyses. Developing consensus guidelines for data preprocessing, feature selection, and model validation is essential for moving beyond the prevailing “tip of the iceberg” interpretations that may misrepresent true neurobiological associations.
